# Functional Outcomes of Intra-articular Distal Radius Fractures: Single-Incision Three-Column Fixation Using Volar Locking Plate and K-Wire Stabilization

**DOI:** 10.7759/cureus.92032

**Published:** 2025-09-11

**Authors:** Sunil Kumar, Harish Kumar, Pradeep K Gupta, Rajendra Kumar, Aman Baijal, Shailendra Yadav, Aman Srivastava, Varun K Verma

**Affiliations:** 1 Orthopaedics and Traumatology, Uttar Pradesh University of Medical Sciences, Etawah, IND; 2 Orthopaedics, Uttar Pradesh University of Medical Sciences, Etawah, IND; 3 Orthopaedic Surgery, Government Institute of Medical Sciences, Greater Noida, IND

**Keywords:** distal end radius fracture, k-wire fixation, quickdash score, single incision, three-column concept, volar locking plate

## Abstract

Background: Distal radius fractures are common, accounting for 17% of all fractures and 75% of forearm fractures. Their age distribution is bimodal, affecting younger individuals with high-energy trauma and older adults with osteoporotic bones. Efficient management is essential to prevent long-term complications such as malunion and chronic discomfort. This study aims to evaluate the clinical and functional outcomes of intra-articular distal radius fractures treated with a modified surgical procedure using a 2.7-mm locking volar plate and K-wires for three-column fixation via a single volar approach.

Methods: This prospective interventional study was conducted at a tertiary care institute from January 2019 to August 2020. Patients with closed or open (Gustilo Anderson Type I) intra-articular distal radius fractures classified as AO Types B and C were included. Exclusion criteria were complex fractures (Type II or III), pathological fractures, or significant comorbidities. Thirty-five patients underwent treatment with volar plating, with or without K-wire fixation, and were subsequently monitored for clinical and functional outcomes using QuickDASH ratings and range of motion assessments. Radiographic evaluations were performed to assess fracture alignment and healing.

Results: The study included 24 males and 11 females, with a mean age of 35.2 years. QuickDASH scores improved significantly, from 67.8 at one month to 8.07 at six months (p < 0.00001). Wrist range of motion also showed marked improvement across all parameters. Radiographic findings remained stable during follow-up, with no changes in volar tilt, radial inclination, or ulnar variance. Complications were minimal, with three patients developing wrist stiffness and one patient experiencing a postoperative infection.

Conclusion: The modified surgical technique using a 2.7-mm locking volar plate and K-wires for three-column fixation offers efficient stabilization and functional recovery in intra-articular distal radius fractures. This method offers significant improvements in wrist function while maintaining consistent radiographic outcomes, supporting its use in the management of complex fractures.

## Introduction

Orthopedic injuries most often involve distal radius fractures [1]. These account for 17% of all fractures and 75% of forearm fractures, underscoring their prevalence. Colles first highlighted their significance [2], and his observations remain relevant today, with current data indicating a 17% lifetime risk of sustaining a distal radius fracture. Distal radius fractures show a clear bimodal age distribution. The first group consists of younger individuals, typically injured through high-energy trauma such as vehicular accidents or sports injuries. The second group includes older adults, especially those with osteoporosis, who sustain fractures from low-energy trauma, such as falls from standing height. This distribution reflects the differing causes of the injury, from high-impact trauma in the young to fragility fractures in the elderly [1-3].

Despite their frequency, distal radius fractures may be overlooked in polytrauma cases, particularly when high-energy injuries such as femoral or pelvic fractures demand immediate attention. In such situations, initial conservative management of the distal radius may lead to suboptimal outcomes. However, achieving and maintaining near-anatomic restoration of the distal radius is essential for preserving wrist function and preventing complications such as malunion, chronic pain, and long-term disability [4].

Management of distal radius fractures has evolved considerably, with greater emphasis on restoring radial length, radial tilt, and articular surface congruity to achieve good functional results. Failure to do so can lead to deformities and disability that impair wrist and hand function. Current treatment options range from non-surgical methods, such as closed reduction and immobilization in a plaster of Paris (POP) cast, to surgical approaches, including percutaneous pin fixation, intra-focal pinning (Kapandji technique), external fixation, and internal fixation with plates [5,6].

A major advance in fracture management is the three-column theory introduced by Rikli and Regazzoni [7]. This model divides the wrist into three columns: radial, intermediate, and ulnar. The radial column consists of the radial styloid and scaphoid fossa, essential for carpal support and ligament attachment. The intermediate column includes the lunate fossa and its articulation with the ulnar head, both vital for carpal alignment. The ulnar column comprises the triangular fibrocartilage complex (TFCC) and the ulnar head, which are crucial for forearm rotation and distal radioulnar joint (DRUJ) stability. This biomechanical understanding has strongly influenced surgical strategies for complex fractures [8].

Volar plating has become the preferred method for open reduction and internal fixation (ORIF) of distal radius fractures, as it provides stable fixation and supports early functional recovery [9]. Compared with dorsal plating, volar plates allow controlled reduction and stabilization while reducing the risk of complications such as extensor tendon irritation or rupture [5]. In certain complex fracture patterns, particularly those with multiple fragments, a fragment-specific approach focusing on individual columns may be more appropriate. This can be performed alone or in combination with volar plating to stabilize fragments not adequately controlled by a single plate [10,11]. This study aimed to evaluate the clinical and functional outcomes of intra-articular distal radius fractures treated with a modified surgical technique designed for three-column fixation through a single volar approach. The method used a 2.7-mm volar locking plate with K-wires to secure the radial and ulnar columns. Developed to address the limitations of conventional approaches, this technique provides comprehensive stabilization of the distal radius by simultaneously targeting the radial, intermediate, and ulnar columns with fragment-specific reduction, in line with current recommendations for complex fracture management.

## Materials and methods

This prospective study was conducted at Uttar Pradesh University of Medical Sciences, Saifai, Etawah, Uttar Pradesh, India, a tertiary care institute in North India, from January 2019 to August 2020. The aim was to evaluate the clinical and functional outcomes of patients with intra-articular distal radius fractures treated with a 2.7-mm volar locking plate and K-wires using a modified surgical technique. Prior to the study, ethical approval was obtained from the institutional ethics committee, and written informed consent was obtained from all participants.

Participants were selected from patients presenting to the emergency department and orthopedic outpatient clinic with intra-articular distal radius fractures that met the study’s inclusion and exclusion criteria. Inclusion criteria were closed or open distal radius fractures up to Gustilo Anderson Type I, radiologically confirmed intra-articular fractures classified as Type B or C according to the AO classification, age between 20 and 60 years, medically fit, and willing to undergo surgery. Exclusion criteria included compound fractures classified as Gustilo Anderson Type II or III, pathological fractures, neurovascular deficits, or medical comorbidities precluding surgical fitness.

The total sample size was 35, as 2 of the 37 patients who met the inclusion criteria were lost to follow-up. Standard emergency care protocols were followed, including wound care, immobilization with a POP slab, supportive care, and management of associated injuries. X-rays were used to confirm the diagnosis and assist in surgical planning. Routine preoperative investigations were carried out, and all patients received pre-anesthetic clearance before being scheduled for elective surgery.

**Figure 1:**
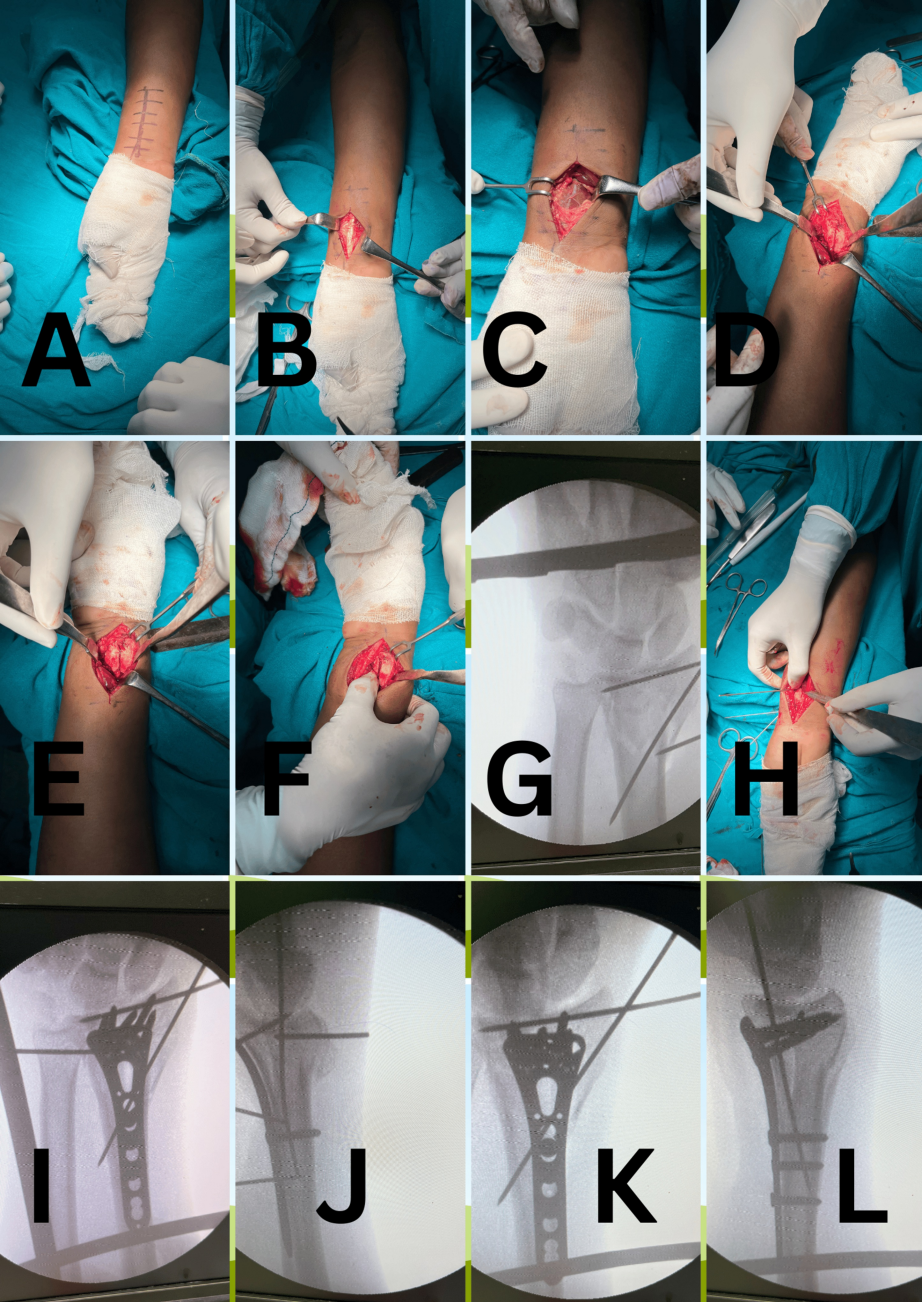
Technique of three-column distal radius fracture fixation with volar locking plate and K-wires through a single incision (A) Patient positioning and incision marking. (B) FCR tendon visible through the incision. (C) Pronator quadratus muscle exposed after retracting the FCR tendon. (D) Radial sigmoid notch and fracture fragments visible through the incision. (E) Middle column fracture visible. (F) Radial column and radial styloid fully exposed. (G) Fracture fragments temporarily fixed with multiple 2.0-2.5-mm K-wires (AP view X-ray). (H) Fracture fragments temporarily fixed with multiple 2.0-2.5-mm K-wires. (I) AP X-ray after volar plate fixation with K-wires. (J) Lateral X-ray after volar plate fixation with K-wires. (K) Final AP X-ray showing volar plate fixation with K-wire in radial styloid and sigmoid notch. (L) Final lateral X-ray showing volar plate fixation with K-wire in radial styloid and sigmoid notch.

During surgery, patients were positioned supine on a radiolucent table, and an image intensifier was used to visualize the distal radius and ulna. Surgery was performed under general or regional anesthesia, with diathermy and a pneumatic tourniquet used for hemostasis. The Modified Henry (trans-FCR) approach was followed [12], involving a straight palmar incision over the flexor carpi radialis (FCR), beginning 5 cm proximal to the wrist joint line. The distal radius was fully exposed, including the radial column (volar and lateral surfaces), the middle column, and the radial sigmoid notch of the DRUJ. Fracture sites were cleaned and mobilized. Fracture fragments were reduced under direct vision, while additional ulnar column injuries were managed indirectly under C-arm guidance. In cases with dorsally displaced fragments or dorsal comminution, the volar fragments were retracted to allow direct visualization and reduction of the dorsal fragments before reducing the volar fragments. All fracture fragments were addressed individually and temporarily fixed with multiple K-wires. When the radial column was involved, the radial styloid was accessed by retracting the radial artery medially and then reduced and stabilized with a 2.0-2.5-mm K-wire. The intermediate column, including the radiocarpal and radioulnar joint surfaces, was anatomically reduced with temporary K-wire fixation and stabilized with a low-profile volar locking plate, using 2.7-mm screws at the metaphysis and 3.5-mm screws at the diaphysis. The plate was positioned distally at the watershed line of the radius to ensure buttressing of the volar lunate corner and the sigmoid notch. DRUJ stability was tested in cases involving the ulnar column or suspected DRUJ disruption. If instability was present, additional percutaneous K-wire fixation was performed. The wound was then closed, and the wrist was immobilized with a below-elbow POP slab (Figure 1).

Postoperatively, patients were encouraged to begin early active finger range of motion exercises as tolerated. Sutures were removed at two weeks, and the POP slab was removed at three weeks, after which patients started passive and active wrist exercises. Of the 35 patients, 25 (71.4%) were treated with volar plate fixation alone. In 10 cases (28.6%), additional K-wire fixation was used: four patients with ulnar column fractures, three with radial styloid fractures (radial column), and three with three-column injuries requiring transfixing K-wires across the radius and ulna. These wires were removed after four weeks, followed by wrist mobilization. The average duration of surgery was 120 minutes (range: 40 minutes to 2 hours), and the mean perioperative blood loss was approximately 100 mL.

Patients were followed up at one, two, three, and six months postoperatively. Functional recovery was assessed at each visit using the QuickDASH score [13], and wrist range of motion (palmar flexion, dorsiflexion, ulnar deviation, radial deviation, pronation, and supination) was measured at the same time points. Radiographic evaluations were performed immediately after surgery and at each follow-up to assess fracture alignment and healing.

All statistical analyses were performed using IBM SPSS Statistics for Windows, Version 20.0 (Released 2011; IBM Corp., Armonk, NY, USA). The primary outcome was the QuickDASH score, while secondary outcomes included wrist range of motion, radiographic findings, and complications. Numerical variables were presented as mean ± standard deviation, and categorical variables as frequencies and percentages. Differences in functional outcomes (mean QuickDASH scores) and range of motion across follow-up periods were analyzed using repeated measures analysis of variance (ANOVA). A p-value of < 0.05 was considered statistically significant.

## Results

This study included 24 male and 11 female participants, aged 20 to 60 years, with a mean age of 35.2 years. Fractures occurred in the right wrist in 21 patients and in the left wrist in 14 patients. Of the 35 patients, 32 (91.4%) presented with closed fractures, while 3 (8.6%) had open fractures classified as Gustilo Anderson Type I. Associated injuries were uncommon: one case each of proximal tibia fracture, clavicle fracture, distal femur fracture, and head injury, each accounting for 2.8% of the study population. The majority of patients (88.6%) had no additional injuries. According to the AO classification, 2.8% of fractures were Type B1, 2.8% were Type B2, 5.6% were Type B3, 31.4% were Type C1, 45.7% were Type C2, and 11.4% were Type C3 (Table 1).

**Table 1:** Demographics of patients included in the study RTA: road traffic accident, DRUJ: distal radioulnar joint.

Variable	Number of cases	Percentages
Age (in years)		
<30	11	31.4%
30-40	14	40%
41-50	9	25.7%
Gender		
Male	24	68.6%
Female	11	31.4%
Side involved		
Left	14	40%
Right	21	60%
Mode of injury		
Fall on outstretch hand	12	34.3%
RTA	23	65.7%
AO classification		
B1	1	2.8%
B2	1	2.8%
B3	2	5.6%
C1	11	31.4%
C2	16	45.7%
C3	4	11.4%
Open/close		
Close	32	91.4%
Open (Gustilo Type I)	3	8.6%
Other associated trauma		
Fracture proximal tibia	1	2.8%
Fracture clavicle	1	2.8%
Fracture distal femur	1	2.8%
Head injury	1	2.8%
No associated injury	1	2.8%
DRUJ injury	31	88.6%
Present	2	5.6%
Absent	33	94.4%
Ulnar head/styloid fracture		
Present	7	20.0%
Absent	28	80.0%
Radial styloid fracture		
Present	3	11.4%
Absent	31	88.6%
Method of fixation		
Volar plate radius alone	25	71.42%
Volar plate radius with K-wire fixation of radial/ulnar columns	10	28.58%

The QuickDASH scores showed marked functional improvement over time. The mean score decreased from 67.8 at one month post-surgery to 32.7 at two months, 18.7 at three months, and 8.07 at six months. This reduction was statistically significant, with an F-value of 4811 and a p-value < 0.00001, indicating substantial functional recovery throughout the follow-up period (Tables 2 and 3).

**Table 2:** Functional outcomes at one, two, three, and six months of follow-up showed a significant decrease in mean QuickDASH scores after surgery A significance level of p < 0.05 was set for all statistical tests, with any p-value below this threshold considered statistically significant. To assess differences in functional outcomes (mean QuickDASH scores) and range of motion across the various follow-up periods, repeated measures analysis of variance (ANOVA) was employed.

QuickDASH at follow-up	Number of cases	Mean ± SD	F-value	p-value
One month	35	67.8 ± 1.9	4811	<0.00001
Two months	35	32.7 ± 3.7		
Three months	35	18.7 ± 4.9		
Six months	35	8.07±4.5		

**Table 3:** Functional QuickDASH score grading in correlation with Green and O’Brien criteria

QuickDASH score	Grade	Number of cases	Percentage
0-8.2	Excellent	25	71.4
8.3-16.9	Good	6	17.14
17-20.7	Fair	3	8.6
>20.7	Poor	1	2.9

**Figure 2:**
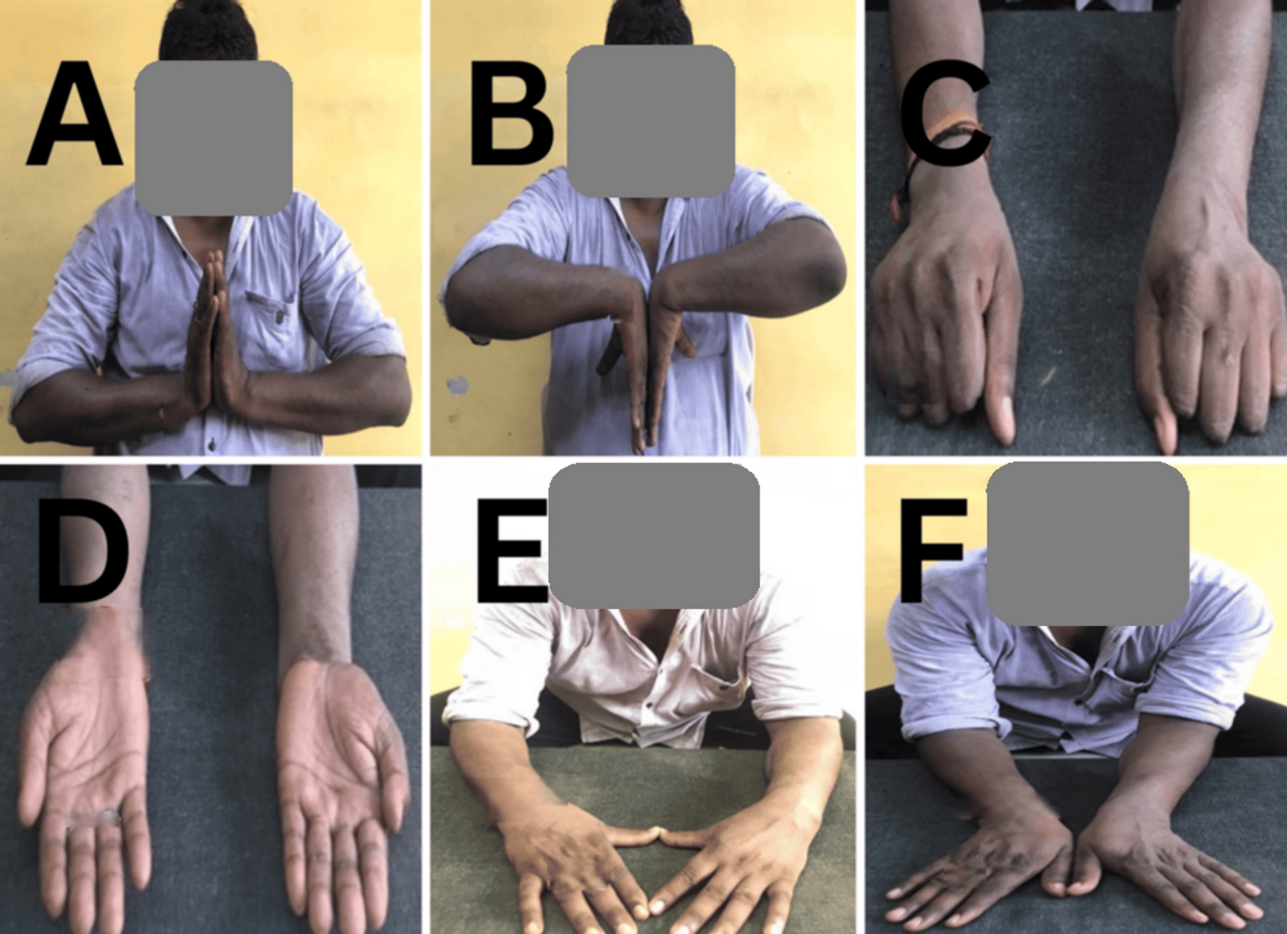
Case 1 clinical photographs at final follow-up (A) Dorsiflexion of the wrist. (B) Palmar flexion. (C) Pronation. (D) Supination. (E) Radial deviation. (F) Ulnar deviation.

**Figure 3:**
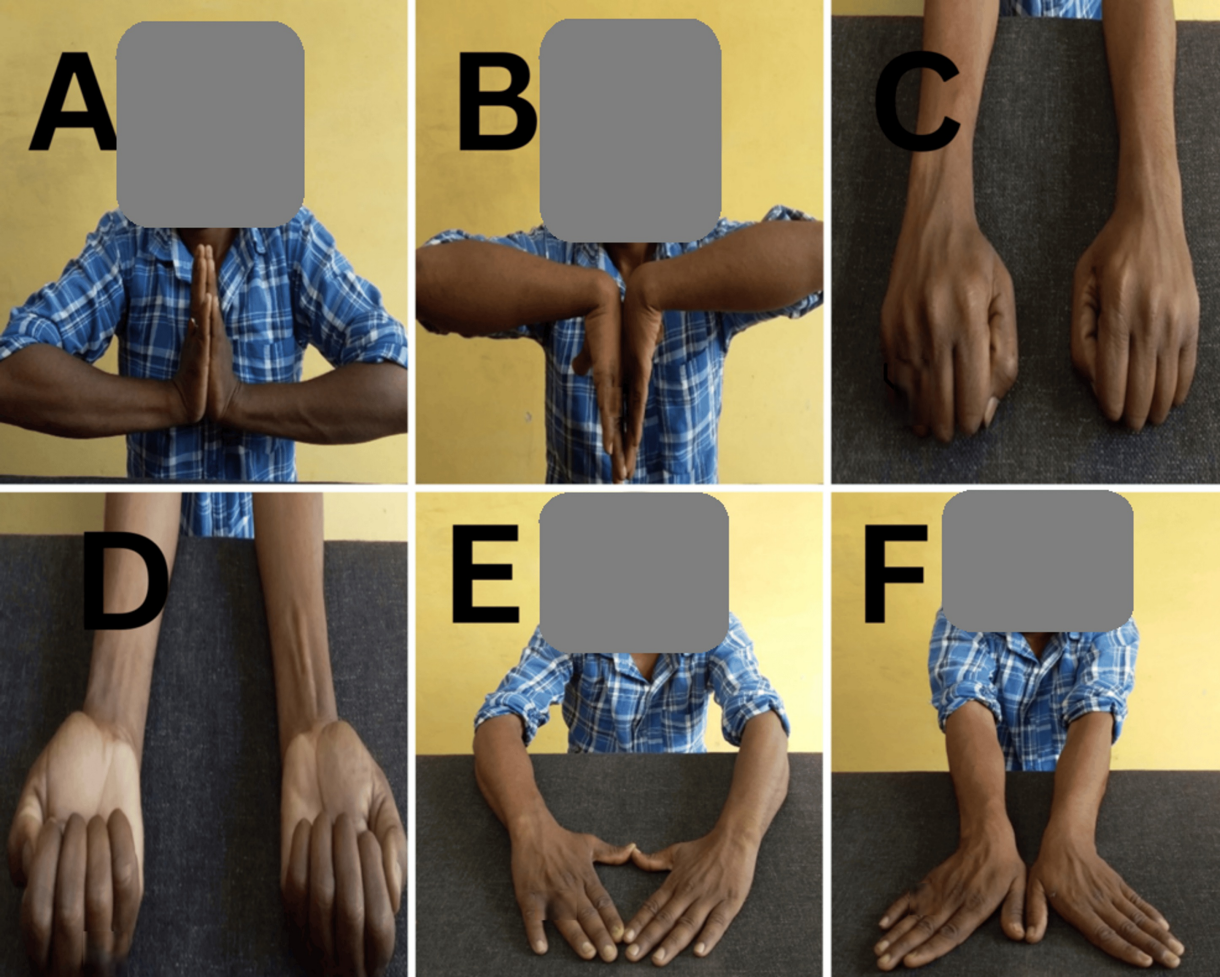
Case 2 clinical photographs at final follow-up (A) Dorsiflexion of the wrist. (B) Palmar flexion. (C) Pronation. (D) Supination. (E) Radial deviation. (F) Ulnar deviation.

**Figure 4:**
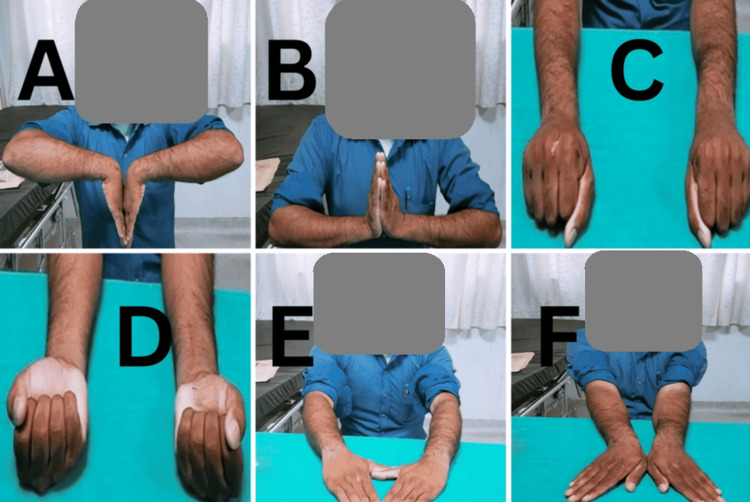
Case 3 clinical photographs at final follow-up (A) Dorsiflexion of the wrist. (B) Palmar flexion. (C) Pronation. (D) Supination. (E) Radial deviation. (F) Ulnar deviation.

Wrist range of motion also improved significantly across all metrics. Palmar flexion increased from an average of 57.6° at one month to 72.5° at six months. Similarly, dorsiflexion improved from 52.4° to 63.3°, supination from 58.6° to 72.6°, pronation from 62.3° to 75.7°, radial deviation from 12.3° to 18.8°, and ulnar deviation from 14.2° to 22.6°. All these improvements were statistically significant, indicating enhanced wrist mobility (Table 4) (Figures 2-4).

**Table 4:** Clinical outcomes based on range of motion at regular follow-ups Repeated measures analysis of variance (ANOVA) was employed. A significance level of p < 0.05 was set for all statistical tests, with any p-value below this threshold considered statistically significant.

Follow-up (in months)	Dorsiflexion (mean ± SD)	Palmar flexion (mean ± SD)	Supination (mean ± SD)	Pronation (mean ± SD)	Radial deviation (mean ± SD)	Ulnar deviation (mean ± SD)
One month	52.4 ± 6.6	57.6 ± 7.6	58.6 ± 7.3	62.3 ± 7.4	12.3 ± 1.7	14.2 ± 2.3
Two months	56.05 ± 6.7	61.6 ± 6.12	64.8 ± 6.6	70.5 ± 6.9	16.8 ± 2.5	18.4 ± 2.2
Three months	58.6 ± 6.03	68.5 ± 4.9	68.5 ± 6.9	73.6 ± 6.9	17.7 ± 2.3	20.5 ± 1.7
Six months	63.3 ± 5.4	72.5 ± 5.5	72.6 ± 7.0	75.7 ± 6.9	18.8 ± 2.1	22.6 ± 2.0
F-value	19.2	41.8	25.1	20.3	59.9	105.4
p-value	<0.00001	<0.00001	<0.00001	<0.00001	<0.00001	<0.00001

**Figure 5:**
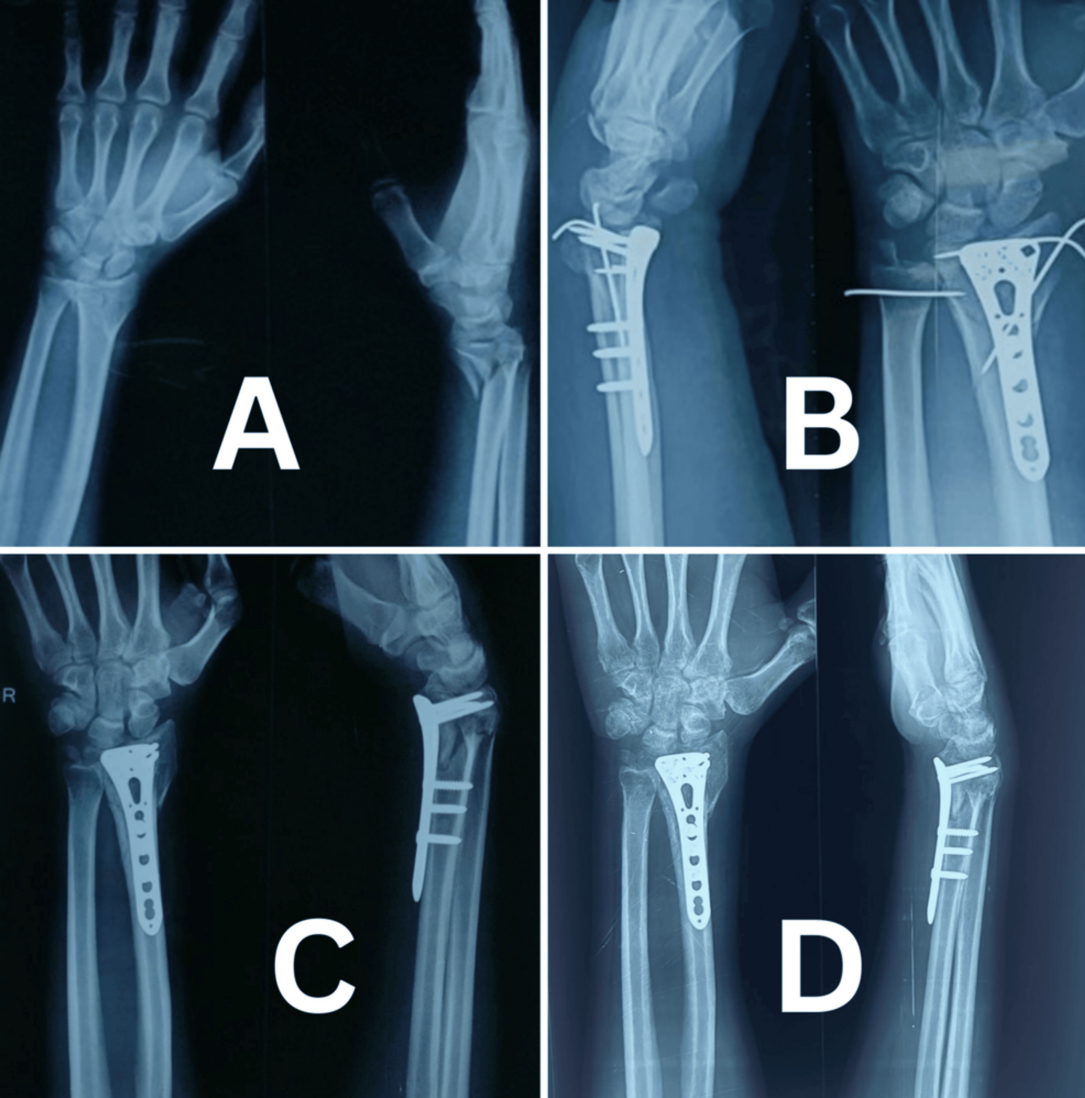
Case 1 wrist X-rays (A) Preoperative anteroposterior and lateral wrist X-rays showing a distal radius fracture. (B) Postoperative anteroposterior and lateral wrist X-rays showing fracture fixation. (C) Three-month follow-up after K-wire removal. (D) Six-month follow-up showing united fracture and congruent joint lines.

**Figure 6:**
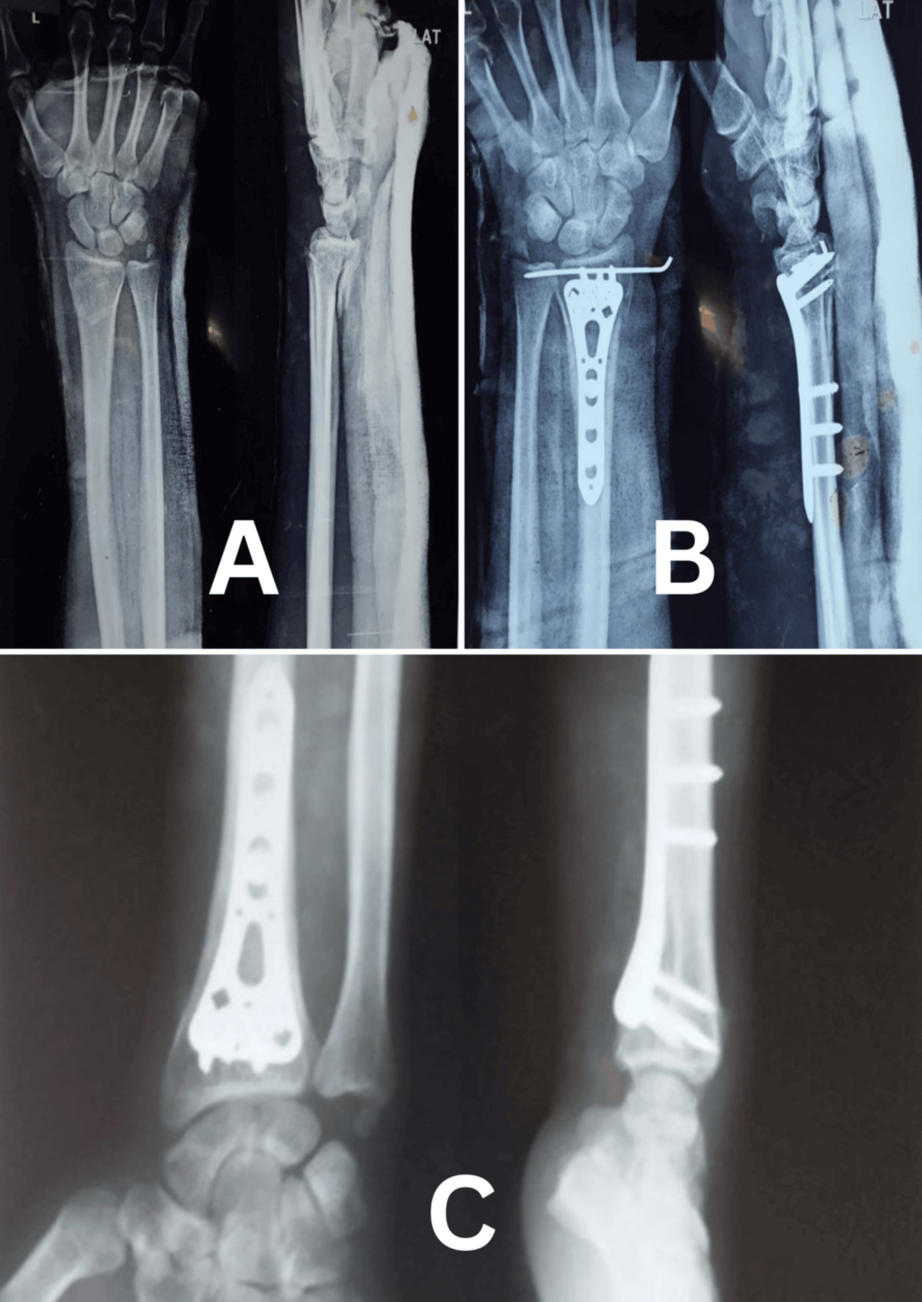
Case 2 wrist X-rays (A) Preoperative anteroposterior and lateral wrist X-rays showing a distal radius fracture. (B) Postoperative anteroposterior and lateral wrist X-rays showing fracture fixation. (C) Six-month follow-up anteroposterior and lateral X-rays showing fracture union.

**Figure 7:**
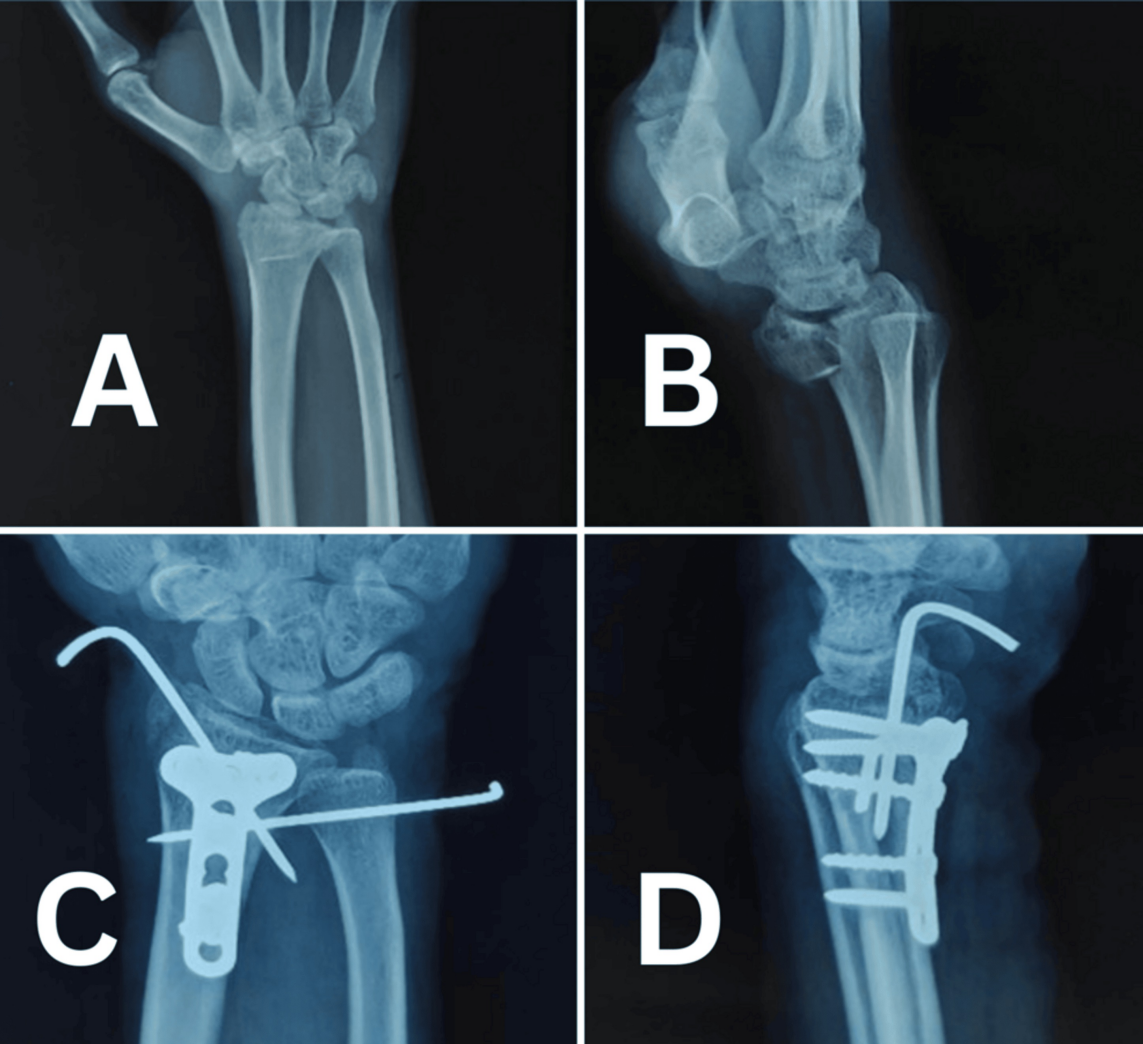
Case 3 wrist X-rays (A) Preoperative anteroposterior wrist X-ray showing a distal radius fracture. (B) Preoperative lateral wrist X-ray showing distal radius fracture. (C) One-month postoperative anteroposterior wrist X-ray showing fracture fixation and early union. (D) Postoperative anteroposterior wrist X-ray showing fracture fixation and union.

Radiographic outcomes remained stable throughout the follow-up period. Mean volar tilt decreased slightly from 10.4° at one month to 9.2° at six months, while radial inclination remained stable around 20°. Radial length showed minimal fluctuations, and ulnar variance remained constant with no significant changes (Table 5 and Table 6) (Figures 5-7).

**Table 5:** Radiological outcomes of patients at different follow-up periods Wrist X-ray angles and lengths were evaluated postoperatively at each follow-up.

Follow-up in months	Volar tilt (mean ± SD)	Radial inclination (mean ± SD)	Radial length (mean ± SD)	Ulnar variance (mean ± SD)
1	10.4 ± 2.30	20.71 ± 3.18	10.81 ± 2.63	-1.29 ± 0.32
2	10.3 ± 2.23	20.62 ± 3.25	10.76 ± 2.60	-1.21 ± 0.48
3	10.0 ± 2.28	20.45 ± 3.42	10.64 ± 2.56	-1.20 ± 0.48
6	9.2 ± 1.98	20.17 ± 3.63	10.48 ± 2.47	-1.19 ± 0.47

**Table 6:** Radiological outcomes of patients at final follow-up Repeated measures analysis of variance (ANOVA) was employed. A significance level of p < 0.05 was set for all statistical tests, with any p-value below this threshold considered statistically significant.

Follow-up in months	Volar tilt			Radial inclination			Radial length			Ulnar variance
At final follow-up	(In degrees)	No. of cases	%	(In degrees)	No. of cases	%	(In mm)	No. of cases	%	
	0-5	4	11.4	10-14	2	5.7	<3	27	77.2	
	6-10	26	74.3	15-19	14	40	3-6	7	20	
	11-15	5	14.2	20-25	19	54.3	>6	1	2.8	
F-value	2.26			0.175			0.11			0.39
p-value	0.08			0.91			0.95			0.75

Complications were minimal in this study. Three patients (8.5%) experienced wrist stiffness, and one patient (2.8%) developed a postoperative wound infection, which was successfully treated. The majority of patients (88.7%) did not experience any complications (Table 7).

**Table 7:** Complications in patients

Complications	Number of cases	Percentage
Wrist stiffness	3	8.5
Infection	1	2.8
No complication	31	88.7

## Discussion

Fractures of the distal radius are common upper limb injuries, and their prevalence is rising worldwide. In younger adults, the increase is largely attributed to road traffic accidents (RTAs), while in older adults, it is linked to the growing ageing population [14]. Severe trauma or advanced osteoporosis often leads to complex fractures with intra-articular extension, significant displacement, and comminution. These complex patterns are difficult to manage conservatively, making surgical intervention more appropriate in many cases [11].

The study cohort consisted of 35 participants, predominantly male, with a mean age of 35.2 years. More than half of the fractures involved the right wrist. A large proportion of cases (88.5%) were classified as AO Type C, reflecting the high prevalence of complex intra-articular fractures. The middle column was affected in all patients, while radial styloid (radial column) fractures occurred in six cases. Additionally, two patients had DRUJ injuries, and five had distal ulna fractures (ulnar column injuries), all of which were surgically stabilized. These demographic and injury patterns are consistent with findings from previous studies, such as those by Kwan et al. [15], and align with the observations reported by Chung et al., Gogna et al., Kiliç et al., Anakwe et al., Catalano III et al., Fitoussi et al., Orbay et al., and Fok et al. [16-23].

Significant progress has been made in the management of intra-articular distal radius fractures over the years. Various techniques, including percutaneous pinning, plating, and external fixation, have been described in the literature [24-26]. The success of these procedures largely depends on restoring key anatomical parameters, such as radial height, palmar tilt, radial inclination, and articular congruity of the radiocarpal joints and DRUJ [26]. 

The introduction of Rikli and Regazzoni’s three-column model of the distal radius and ulna in 1996 greatly advanced the understanding of wrist biomechanics and improved fracture management [7]. Subsequent research has led to the development of fragment-specific fixation techniques, in which each fracture fragment is addressed separately according to the complexity of the injury [10,11]. Stabilizing such fractures often requires multiple implants and incisions to restore the integrity of all columns. Jacobi et al. described a radio-volar double plating procedure using a single Henry approach [27], while Kotsalis et al. demonstrated the use of the single trans-FCR technique to achieve three-column fixation [28]. In the present study, fixation was achieved using a 2.7-mm low-profile volar plate and K-wires, applied in a fragment-specific manner according to the three-column principle. All procedures were performed through a single modified Henry incision, a safe approach that minimizes the risk of radial artery or median nerve injury. This technique offers excellent exposure, allowing precise alignment of the radial and middle columns and direct visualization of the DRUJ in the ulnar column. Temporary stabilization of all fracture fragments, including dorsal fragments, was achieved with multiple K-wires, ensuring accurate anatomical reduction in every case.

According to Bachoura and Shin [25], the heterogeneity of distal radius fractures makes it difficult to rely on a single implant, surgical method, or fixation system. In this study, however, we chose a single-incision approach. Unlike earlier studies that used plates to stabilize both the radial and ulnar columns, which require a larger surface area, we used 2.5-mm K-wires for fixation after proper alignment of the fragments. Ulnar column injuries were managed with percutaneous K-wire fixation under direct visualization of the DRUJ, with successful reduction confirmed by the alignment of the sigmoid notch [28]. While K-wires alone may not provide ideal stability, the primary stabilization in our method was achieved using a volar locking plate, with K-wires serving as an adjunct for fragment-specific fixation. Vipin et al. reported functional success with a new percutaneous technique using five pins for distal radius fractures, highlighting the stability achieved with multiple K-wires [29]. Similarly, Żyluk et al. found that K-wire fixation provided favorable outcomes in distal radius fractures [30].

Volar plates are widely used because they provide reliable stabilization and support for both the middle and radial columns of the wrist [31]. The middle column is the primary weight-bearing area, transmitting axial loads through the wrist, and stabilizing the lunate facet is critical to prevent carpal subluxation or dislocation [32]. However, 3.5-mm locking volar plates placed at the watershed line can cause tendon irritation and other hardware-related complications, while positioning them proximally may compromise fixation of the lunate facet. To minimize these risks, we used 2.7-mm low-profile locking plates positioned at the watershed line, as recommended by Kotian et al. [33]. These plates allow subchondral fixation with minimal soft-tissue irritation, making them especially suitable for comminuted fractures and osteoporotic bone [34].

The findings of our study are consistent with previous research, confirming the effectiveness of volar plate fixation in restoring function and achieving stable radiographic outcomes. Similar results were reported by Kotian et al. [33], Rozental et al. [35], and Konstantinidis et al. [36]. In our series, QuickDASH scores showed a marked improvement, decreasing from 67.8 at one month to 8.07 at six months, in line with outcomes reported in earlier studies on volar plating. The improvement in wrist function was statistically significant across all parameters of range of motion, including palmar flexion, dorsiflexion, pronation, supination, radial deviation, and ulnar deviation. Radiographic results also remained stable throughout follow-up, with only minor variations in volar tilt, radial inclination, and ulnar variance. This sustained stability demonstrates that fracture reduction achieved at surgery was well maintained during the postoperative period. These findings are consistent with the results of Egol et al. [37], Rozental et al. [38], Rizzo et al. [39], and Gangadhar et al. [40].

While volar plating is currently the preferred technique for distal radius fractures, fragment-specific and column-based fixation remain important for managing complex patterns [8]. Fragment-specific plates have their own advantages and disadvantages [9]. Several studies have compared fragment-specific fixation (FSF) with volar locking plate systems (VLPS), showing that volar plates generally provide superior fixation and functional outcomes [41-44]. Sammer et al. compared FSF with VLPS and concluded that VLPS offers more stable fixation and better early postoperative outcomes, while FSF is associated with a higher rate of complications requiring reoperation [34]. The literature suggests that fragment-specific plates can be a useful supplement to volar plating. In our study, however, we used K-wires in place of fragment-specific plates to achieve fragment-targeted fixation. This approach minimized the need for additional implants and incisions, while still providing sufficient stabilization. Our results show that a single-incision technique can achieve adequate exposure for three-column and fragment-specific fixation. Similar to the approaches described by Jacobi et al. [27] and Kotsalis et al. [28], our method combined K-wires with a volar locking plate, producing excellent outcomes without complications or the need for reoperation.

## Conclusions

This study has several limitations, including a small sample size, short follow-up duration, and the absence of both a control group and a comparative cohort. The study population also consisted mainly of young adults with high-energy trauma, resulting in a relatively uniform group in terms of age and injury pattern. In most cases, volar plating alone was used to stabilize the radial and ulnar columns, while K-wires were applied in only 10 cases, including just three with three-column injuries. Therefore, the effectiveness of K-wire fixation may vary depending on fracture complexity.

The combined use of a 2.7-mm volar locking compression plate (LCP) and K-wires for three-column fixation of distal radius fractures demonstrated excellent functional and radiological outcomes with minimal complications. This technique offers orthopedic surgeons a reliable option for managing complex intra-articular fractures. Future research comparing this approach with other fragment-specific techniques, particularly those requiring multiple incisions, may provide further guidance for optimizing treatment of challenging distal radius fractures.

## References

[1] Liporace FA, Adams MR, Capo JT, Koval KJ (2009). Distal radius fractures. J Orthop Trauma.

[2] Colles A (1814). On the fracture of the carpal extremity of the radius. Edinb Med Surg J.

[3] Knirk JL, Jupiter JB (1986). Intra-articular fractures of the distal end of the radius in young adults. J Bone Joint Surg Am.

[4] Bassett RL (1987). Displaced intraarticular fractures of the distal radius. Clin Orthop Relat Res.

[5] Jakubietz MG, Gruenert JG, Jakubietz RG (2012). Palmar and dorsal fixed-angle plates in AO C-type fractures of the distal radius: is there an advantage of palmar plates in the long term?. J Orthop Surg Res.

[6] Ruch DS, Papadonikolakis A (2006). Volar versus dorsal plating in the management of intra-articular distal radius fractures. J Hand Surg Am.

[7] Rikli DA, Regazzoni P (1996). Fractures of the distal end of the radius treated by internal fixation and early function. A preliminary report of 20 cases. J Bone Joint Surg Br.

[8] Hoekzema NA, Brambila M (2021). Column-specific distal radius fracture fixation. J Orthop Trauma.

[9] Emet A, Veizi E, Karaman Y, Akgun E, Tolunay T, Firat A (2024). Volar fixed plating of distal radius fractures: optimizing plate position for enhanced clinical outcomes. BMC Musculoskelet Disord.

[10] Hozack BA, Tosti RJ (2019). Fragment-specific fixation in distal radius fractures. Curr Rev Musculoskelet Med.

[11] Geissler WB, Clark SM (2016). Fragment-specific fixation for fractures of the distal radius. J Wrist Surg.

[12] Shim BJ, Kim DY, Lee SS, Cho MS, Hwang JT (2022). Comparison of the conventional Henry approach and trans-flexor carpi radialis approach for the treatment of distal radius fracture: a retrospective cohort study. Medicine (Baltimore).

[13] Hudak PL, Amadio PC, Bombardier C (1996). Development of an upper extremity outcome measure: the DASH (disabilities of the arm, shoulder and hand) [corrected]. The Upper Extremity Collaborative Group (UECG). Am J Ind Med.

[14] Kim HJ, Lee HJ, Kim H, Cho SW, Kim JS (2009). Targeted genome editing in human cells with zinc finger nucleases constructed via modular assembly. Genome Res.

[15] Kwan K, Lau TW, Leung F (2011). Operative treatment of distal radial fractures with locking plate system-a prospective study. Int Orthop.

[16] Chung KC, Watt AJ, Kotsis SV, Margaliot Z, Haase SC, Kim HM (2006). Treatment of unstable distal radial fractures with the volar locking plating system. J Bone Joint Surg Am.

[17] Gogna P, Selhi HS, Singla R, Devgan A, Magu NK, Mahindra P, Yamin M (2013). Dorsally comminuted fractures of the distal end of the radius: osteosynthesis with volar fixed angle locking plates. ISRN Orthop.

[18] Kiliç A, Kabukçuoğlu Y, Ozkaya U, Gül M, Sökücü S, Ozdoğan U (2009). Volar locking plate fixation of unstable distal radius fractures. Acta Orthop Traumatol Turc.

[19] Anakwe R, Khan L, Cook R, McEachan J (2010). Locked volar plating for complex distal radius fractures: Patient reported outcomes and satisfaction. J Orthop Surg Res.

[20] Catalano LW 3rd, Cole RJ, Gelberman RH, Evanoff BA, Gilula LA, Borrelli J Jr (1997). Displaced intra-articular fractures of the distal aspect of the radius. Long-term results in young adults after open reduction and internal fixation. J Bone Joint Surg Am.

[21] Fitoussi F, Ip WY, Chow SP (1997). Treatment of displaced intra-articular fractures of the distal end of the radius with plates. J Bone Joint Surg Am.

[22] Orbay J, Badia A, Khoury RK, Gonzalez E, Indriago I (2004). Volar fixed-angle fixation of distal radius fractures: the DVR plate. Tech Hand Up Extrem Surg.

[23] Fok MW, Klausmeyer MA, Fernandez DL, Orbay JL, Bergada AL (2013). Volar plate fixation of intra-articular distal radius fractures: a retrospective study. J Wrist Surg.

[24] Obert L, Rey PB, Uhring J (2013). Fixation of distal radius fractures in adults: a review. Orthop Traumatol Surg Res.

[25] Bachoura A, Shin EK (2019). Emerging technologies in distal radius fracture fixation. Curr Rev Musculoskelet Med.

[26] Pennington E, Bell S, Hill JE (2023). Should video laryngoscopy or direct laryngoscopy be used for adults undergoing endotracheal intubation in the pre-hospital setting? A critical appraisal of a systematic review. J Paramed Pract.

[27] Jacobi M, Wahl P, Kohut G (2010). Repositioning and stabilization of the radial styloid process in comminuted fractures of the distal radius using a single approach: the radio-volar double plating technique. J Orthop Surg Res.

[28] Kotsalis G, Kotsarinis G, Ladogianni M, Fandridis E (2023). Three column fixation through a single incision in distal radius fractures. J Wrist Surg.

[29] Vipin R, Rengarajan N, Manoharan M, Kesavan K (2021). A novel 5-pin fixation for distal radius fractures and its functional assessment. Journal of Orthopedics and Joint Surgery.

[30] Żyluk A, Skala K, Szlosser Z (2018). A comparison of outcomes of K-wire vs plate fixation for distal radial fractures with regard to patients' quality of life. Acta Orthop Belg.

[31] Huang YM, Chen CY, Lin KC, Tarng YW, Liao CY, Chang WN (2022). Functional outcomes following fixation of a marginal distal radius fracture with two commonly used volar locking plates: a retrospective cohort study. BMC Musculoskelet Disord.

[32] Zhou J, Tang W, Li D, Wu Y (2019). Morphological characteristics of different types of distal radius die-punch fractures based on three-column theory. J Orthop Surg Res.

[33] Kotian P, Mudiganty S, Annappa R, Austine J (2017). Radiological outcomes of distal radius fractures managed with 2.7mm volar locking plate fixation-a retrospective analysis. J Clin Diagn Res.

[34] Sammer DM, Fuller DS, Kim HM, Chung KC (2008). A comparative study of fragment-specific versus volar plate fixation of distal radius fractures. Plast Reconstr Surg.

[35] Rozental TD, Blazar PE (2006). Functional outcome and complications after volar plating for dorsally displaced, unstable fractures of the distal radius. J Hand Surg Am.

[36] Konstantinidis L, Helwig P, Strohm PC, Hirschmüller A, Kron P, Südkamp NP (2010). Clinical and radiological outcomes after stabilisation of complex intra-articular fractures of the distal radius with the volar 2.4 mm LCP. Arch Orthop Trauma Surg.

[37] Egol K, Walsh M, Tejwani N, McLaurin T, Wynn C, Paksima N (2008). Bridging external fixation and supplementary Kirschner-wire fixation versus volar locked plating for unstable fractures of the distal radius. A randomised, prospective trial. J Bone Joint Surg Br.

[38] Rozental TD, Blazar PE, Franko OI, Chacko AT, Earp BE, Day CS (2009). Functional outcomes for unstable distal radial fractures treated with open reduction and internal fixation or closed reduction and percutaneous fixation. A prospective randomized trial. J Bone Joint Surg Am.

[39] Rizzo M, Katt BA, Carothers JT (2008). Comparison of locked volar plating versus pinning and external fixation in the treatment of unstable intraarticular distal radius fractures. Hand (N Y).

[40] Gangadhar G, Khan A, Habib SD, Pandey A, Verma A, Nirala SK, Dave PK (2019). A prospective study on functional evaluation of volar locking plate fixation of distal radius fractures. IOSR-JDMS.

[41] Grindel SI, Wang M, Gerlach M, McGrady LM, Brown S (2007). Biomechanical comparison of fixed-angle volar plate versus fixed-angle volar plate plus fragment-specific fixation in a cadaveric distal radius fracture model. J Hand Surg Am.

[42] Taylor KF, Parks BG, Segalman KA (2006). Biomechanical stability of a fixed-angle volar plate versus fragment-specific fixation system: cyclic testing in a C2-type distal radius cadaver fracture model. J Hand Surg Am.

[43] Cooper EO, Segalman KA, Parks BG, Sharma KM, Nguyen A (2007). Biomechanical stability of a volar locking-screw plate versus fragment-specific fixation in a distal radius fracture model. Am J Orthop (Belle Mead NJ).

[44] Landgren M, Abramo A, Geijer M, Kopylov P, Tägil M (2017). Fragment-specific fixation versus volar locking plates in primarily nonreducible or secondarily redisplaced distal radius fractures: a randomized controlled study. J Hand Surg Am.

